# Association between mitochondrial DNA levels and depression: a systematic review and meta-analysis

**DOI:** 10.1186/s12888-023-05358-8

**Published:** 2023-11-22

**Authors:** Wenhui Li, Lingqun Zhu, Yi Chen, Yudi Zhuo, Shurun Wan, Rongjuan Guo

**Affiliations:** 1 Dongzhimen Hospital, Beijing University of Chinese Medicine, Beijing, 100700 China; 2 Key Laboratory of Chinese Internal Medicine of Ministry of Education and Beijing Key Laboratory of Dongzhimen Hospital, Beijing University of Chinese Medicine, Beijing, 100700 China; 3 Dongfang Hospital, Beijing University of Chinese Medicine, Beijing, 100078 China

**Keywords:** Depression, mtDNA, Mitochondria, Meta-analysis

## Abstract

**Background:**

Mitochondrial dysfunction leading to disturbances in energy metabolism has emerged as one of the risk factors in the pathogenesis of depression. Numerous studies have identified alterations in the content of mitochondrial DNA (mtDNA) in peripheral blood and cerebrospinal fluid of individuals with depression. Researchers have sought to establish a clear association between mtDNA and depression. Consequently, we conducted a comprehensive meta-analysis to assess the existing evidence regarding the impact of mtDNA on depression.

**Methods:**

This study conducted a thorough search of the following databases up to March 13, 2023: PubMed, Embase, the Cochrane Library, the Web of Science, Wanfang Database, SINOMED, the China Science and Technology Journal Database, and China National Knowledge Infrastructure. The meta-analysis was carried out using RevMan (version 5.4) and Stata (version 16.0) software. In addition, publication bias was assessed with funnel plots, Begg’s test and Egger’s test.

**Results:**

Our analysis included data from 10 articles, including 12 studies for further examination. A total of 1400 participants were included in this study, comprising 709 (including 300 males and 409 females) patients with depression and 691 (including 303 males and 388 females) healthy controls. The average age of depressed patients was (42.98 ± 2.55) years, and the average age of healthy people was (41.71 ± 2.6) years. The scales used to assess outcomes are Hamilton-rating scale for Depression(4 articles), Montgomery-Asberg Depression Rating Scale(3 articles), and Mini-Internatioal Neuropsychiatric Interview (1 articles). The meta-analysis revealed significantly higher levels of mtDNA in circulating blood samples and skin fibroblasts of individuals with depression in comparison to healthy controls [standardized mean difference(SMD) = 0.42, 95% confidence intervals(CI): 0.16, 0.67].

**Conclusions:**

Our study concludes that there is a significant (p < 0.05) increase in mtDNA levels in serum, plasma, and cerebrospinal fluid in individuals with depression. These findings suggest that mtDNA could serve as a potential biomarker for diagnosing depression.

**Registration number:**

PROSPERO CRD42023414285.

**Supplementary Information:**

The online version contains supplementary material available at 10.1186/s12888-023-05358-8.

## Introduction

Depression is a heterogenous disorder characterized by symptoms spanning various domains of emotion and behavior, including but not limited to, changes in mood, memory impairment, anhedonia, insomnia, fatigue, reduced appetite and libido, and in severe instances, self-harm and suicide [1]. It poses a significant threat to both mental and physical well-being and stands as the primary cause of global disability [2]. Depression is pervasive and frequently recurrent, with a worldwide prevalence of 4.4% [3]. In China, the lifetime prevalence of depression stands at 6.8%, with major depression accounting for 3.4% [4]. However, the incidence of depression continues to rise annually due to mounting societal, lifestyle, and academic pressures. A substantial proportion of patients fail to achieve remission after undergoing treatment with a selective serotonin reuptake inhibitor, with only 25–27% achieving remission following subsequent antidepressant treatments, and as many as 40% becoming treatment-resistant [5]. An impediment to effective depression care is the inaccurate diagnosis of individuals affected by the condition [6]. Presently, the assessment of depression relies on medical history, clinical symptoms, and specific evaluation scales, with a dearth of objective biomarkers for diagnostic purposes [7]. Thus, the quest for biomarkers to facilitate accurate depression diagnosis is a pressing scientific challenge.

The biological mechanisms underpinning depression remain unknown. Research into depression has expanded beyond conventional hypotheses concerning monoaminergic neurotransmitters and inflammatory mechanisms. An increasing focus has shifted towards examining the interplay between mitochondrial energy metabolism and depression. Depressed individuals often exhibit modifications in inflammatory markers, mitochondrial membrane depolarization, oxidized mtDNA, and thus increased levels of both central and peripheral reactive oxygen species (ROS) [8]. Mitochondria serve as the cellular “energy factories” of cells, preserving cellular stability by modulating calcium homeostasis, contributing to ROS and regulating apoptosis. Nonetheless, malfunctioning mitochondria lead to increased production of mitochondrial ROS (mtROS) and the release of cell-free mtDNA release [9]. MtDNA, as the genetic material of mitochondria, plays a role in mediating mitochondrial energy metabolism by encoding vital proteins required for the assembly and operation of mitochondrial respiratory complexes [10]. The mtDNA copy number can serve as an indicator of mitochondrial function and the extent of mtDNA damage [11].

Given the mounting evidence implicating mitochondrial dysfunction as a prospective molecular factor in depression, we conducted a systematic review and meta-analysis to examine the available literature that investigated the association between mtDNA and depression. To our knowledge, this represents the first study to integrate global data in elucidating the connection between mtDNA and depression.

## Methods

### Study registration

This systematic review and meta-analysis adhered to the Preferred Reporting Items for Systematic Reviews and Meta-Analysis (PRISMA) guidelines [12]. The review protocol was registered with PROSPERO under the registration CRD42023414285.

### Literature search strategy

We conducted a comprehensive search of four English electronic databases and four Chinese literature databases, spanning from their inception to March 13, 2023. The databases included PubMed, EMBASE, Cochrane Library, Web of Science, Wanfang Database, SINOMED, VIP Database, and CNKI. There were no language restrictions imposed in our search. Our search strategy employed the following keywords: (“mtDNA” OR “mitochondrial DNA”) AND (“depression” OR “depressive disorder”). To ensure comprehensive coverage, we also scrutinized the references of all the reviewed publications. Two independent reviewers (WL and YZ) were responsible for this process, and in the event of any disagreement, a third investigator (LZ) was consulted. Furthermore, we scrutinized the references of the papers included, full texts, and bibliographies of all potential articles, including relevant reviews and meta-analyses, in order to identify any additional eligible studies.

### Inclusion and exclusion criteria

The inclusion criteria were established as follows: (a) the study involved individuals with depression; (b) cross-sectional, case–control or longitudinal study; (c) the studies reported mtDNA levels, including mean (M), standard deviation (SD), and sample size; (d) both individuals with depression (cases) and healthy participants (controls) were included in the studies; and (e) in cases where the same datasets were discovered during the search process, only the paper with more comprehensive findings was included in our meta-analysis.

Studies were excluded if they met any of the following criteria: (a) duplications; (b) absence of mtDNA measurement in humans; (c) absence of a healthy control group; (d) constituted case reports, review articles, systematic reviews, meta-analyses, commentaries, editorials, or meeting abstracts; or (e) were in vivo or in vitro studies.

### Data extraction

We used NoteExpress software was used for literature management. Two reviewers (WL and YZ) independently extracted all data, and any disagreements were resolved through discussion, with the involvement of a third person (LZ) when necessary. We recorded pertinent study information, including the first author, publication year, participant characteristics, sample size, sample source, region, and mtDNA contents.

### Quality assessment

Two authors (YC and SW) independently evaluated the risk of bias and methodological quality of the included studies using the Newcastle-Ottawa Scale (NOS), which is an evidence-based quality assessment tool designed for systematic reviews of prospective cohort studies [13]. This scale comprises eight items, and the maximum attainable score is 9 points. Studies scoring ≥ 6 points were deemed of high quality [14].

### Statistical analysis

Cochrane Collaboration software (RevMan 5.4) and Stata (version 16.0) were used for all data analyses. Given the varied units of measurement for mtDNA concentrations (units/μL, copies/μL, C/μL, and some articles not specifying units) in the analyzed reports, the effect size was estimated using the Standardized Mean Difference (SMD). Heterogeneity among the studies was assessed using the I^2^ statistic. I^2^ values within the ranges of 0–25%, 26–50%, 51–75%, and 75–100% were categorized as indicating no, low, moderate, and substantial heterogeneity, respectively [15, 16]. Subgroup analyses were conducted to identify potential sources of heterogeneity, considering specimen type (plasma or non-plasma), detection method (quantitative reverse transcription polymerase chain reaction (qRT-PCR), quantitative polymerase chain reaction (qPCR), or real-time fluorescence polymerase chain reaction (rt-PCR)), and location (Asia, Europe, or North America). Sensitivity analyses were performed to ensure the reliability of the results. Furthermore, Egger’s test and Begg’s test were utilized to investigate publication bias. A z-score was employed to assess effect sizes, and a p-value of < 0.05 was considered statistically significant. All statistical tests were two-tailed, with a 95% CI, and the significance level was set at *P* < 0.05.

## Results

### Study selection

**Fig. 1:**
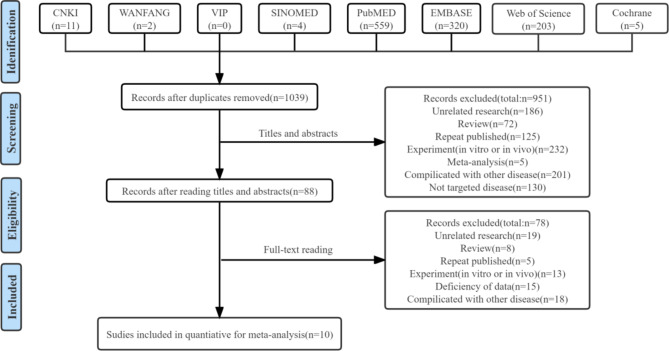
Flow diagram of study selection

The search across the eight databases yielded a total of 1,104 articles for further assessment (11 from CNKI, 2 from Wanfang, 0 from VIP, 4 from SINOMED, 559 from PubMed, 320 from EMBASE, 203 from the Web of Science, and 5 from the Cochrane Library). After reviewing the articles, 65 were excluded due to duplications. Following the examination of titles and abstracts, 951 articles were excluded for various reasons. Ultimately, only ten studies [17–26] met our inclusion criteria after a thorough review of the full texts. The flow diagram is depicted in Fig. 1.

### Study characteristics and quality assessment

**Table 1:** Characteristics of studies included in the present meta-analysis

ID	First author	Year	location	Sample size		Age of DepressionMean(SD)	Age of ControlsMean(SD)	Gender of Depression(M/F)	Gender of Controls (M/F)	Specimen	Detection Method	Diagnostic method
				Depression	Controls							
1	Karen M Ryan [17]	2023	Ireland	100	89	56.36 (14.28)	53.42 (10.39)	38/62	30/59	whole blood	qRT-PCR	HDRS
2a	Emi Ampo [18]	2022	USA	9	16	67.7 (6.5)	69.9 (7.6)	2/7	8/8	Plasma	qRT-PCR	MADRS
2b	Emi Ampo [18]	2022	USA	23	5	70.6 (10.3 )	68.2 (6.9 )	7/16	2/3	Plasma	qRT-PCR	MADRS
3	Vanessa F Gonçalves [19]	2021	Canada	32	21	68.3 (6.7)	70.1 (8.1)	9/23	10/11	Plasma	qRT-PCR	MADRS
4	Johan Fernström [20]	2021	Sweden	47	11	35.0 (10.7)	35.1 (11.1)	21/26	5/6	Plasma	qRT-PCR	MADRS
5	Jae Kyung Chung [21]	2019	Republic of Korea	118	116	47.6 (16.7)	47.7 (16.8)	46/72	46/70	peripheral blood	qPCR	NR
6	Kerstin Kuffner [22]	2020	Germany	16	16	31 (3.12)	32 (2.81)	11/5	11/5	Skin Fibroblasts	qRT-PCR	HDRS
7a	Daniel Lindqvist [23]	2018	Sweden	50	55	39.6 (14.7)	37.6 (13.9)	23/27	22/33	Plasma	rt-PCR	HDRS
7b	Daniel Lindqvist [23]	2018	Sweden	50	55	39.6 (14.7)	37.6 (13.9)	23/27	22/33	PBMCs	rt-PCR	HDRS
8	Cheng Chen Chang [24]	2015	China	40	70	42.00 (18.75)	38.00 (16.50)	12/28	28/42	Leukocyte	rt-PCR	MINI
9	Ying He [25]	2014	China	210	217	30.2 (8.10)	30.8 (7.05)	100/110	109/108	Leukocyte	qPCR	HDRS
10	Kato T [26]	1997	Japan	14	20	48.2 (17.7)	61.2 (10.6)	8/6	10/10	Leukocyte	qPCR	NR

**Abbreviations**: M, Male; F, Female; HDRS, Hamilton-rating scale for Depression; MADRS, Montgomery-Asberg Depression Rating Scale; MINI, Mini-Internatioal Neuropsychiatric Interview. NR, No Report

In total, we included 10 articles covering studies conducted from 1997 to 2023, reporting on 12 studies. These studies involved 709 individuals with depression (300 males and 409 females) and 691 healthy controls (303 males and 388 females). The average age of depressed patients was (42.98 ± 2.55) years, and the average age of healthy people was (41.71 ± 2.6) years. Four articles assessed for depression using the Hamilton-rating scale for Depression (HDRS). Three articles used the Montgomery-Asberg Depression Rating Scale (MADRS). One article used Mini-Internatioal Neuropsychiatric Interview (MINI). The other two articles did not report assessment scales. Most trials were conducted in Asia (2 in China, 1 in Japan, and 1 in Korea) and Europe (2 in Sweden, 1 in Ireland, 1 in Germany). Additionally, trials were carried out in North America (1 in the United States and 1 in Canada). The quantification of mtDNA levels was achieved using quantitative reverse transcription polymerase chain reaction (qRT-PCR), real-time fluorescence polymerase chain reaction (rt-PCR), or quantitative polymerase chain reaction (qPCR). Detailed characteristics of the included studies are presented in Table 1. For the quality evaluation of the included studies, we utilized the Newcastle-Ottawa Scale (NOS), as presented in Table 2. This assessment revealed that the quality of most articles was relatively high, with scores ranging from 5 to 7. Only one study, conducted by Kato T et al. [26], scored five due to a lack of provided information.

**Table 2:** Quality assessment of studies

Study source	Selection				Comparability Comparability of cohorts on the basis of the design or analysis	Exposure			Total score
	Representativeness of the exposed cohort	Selection of the non- exposed cohort	Ascertainment of exposure	Demonstration that outcome of interest was not present at start of study		Assessment of outcome	Was follow-up long enough for outcomes to occur	Adequacy of follow- up of cohorts	
Karen M Ryan [17]	1	1	1	1	2	1	0	0	7
Emi Ampo [18]	1	1	1	1	2	1	0	0	7
Vanessa F Gonçalves [19]	1	1	1	1	2	1	0	0	7
Johan Fernström [20]	1	1	1	1	2	1	0	0	7
Jae Kyung Chung [21]	1	1	1	1	2	1	0	0	7
Kerstin Kuffner [22]	1	1	1	1	2	1	0	0	7
Daniel Lindqvist [23]	1	1	1	1	2	1	0	0	7
Cheng Chen Chang [24]	1	1	1	1	2	1	0	0	7
Ying He [25]	1	1	1	1	1	1	0	0	6
Kato T [26]	1	1	1	0	1	1	0	0	5

A study could be awarded a maximum of 1 point for each item except for the item Comparability of cohorts on the basis of the design or analysis (2 points)

### MtDNA levels in depression

**Fig. 2:**
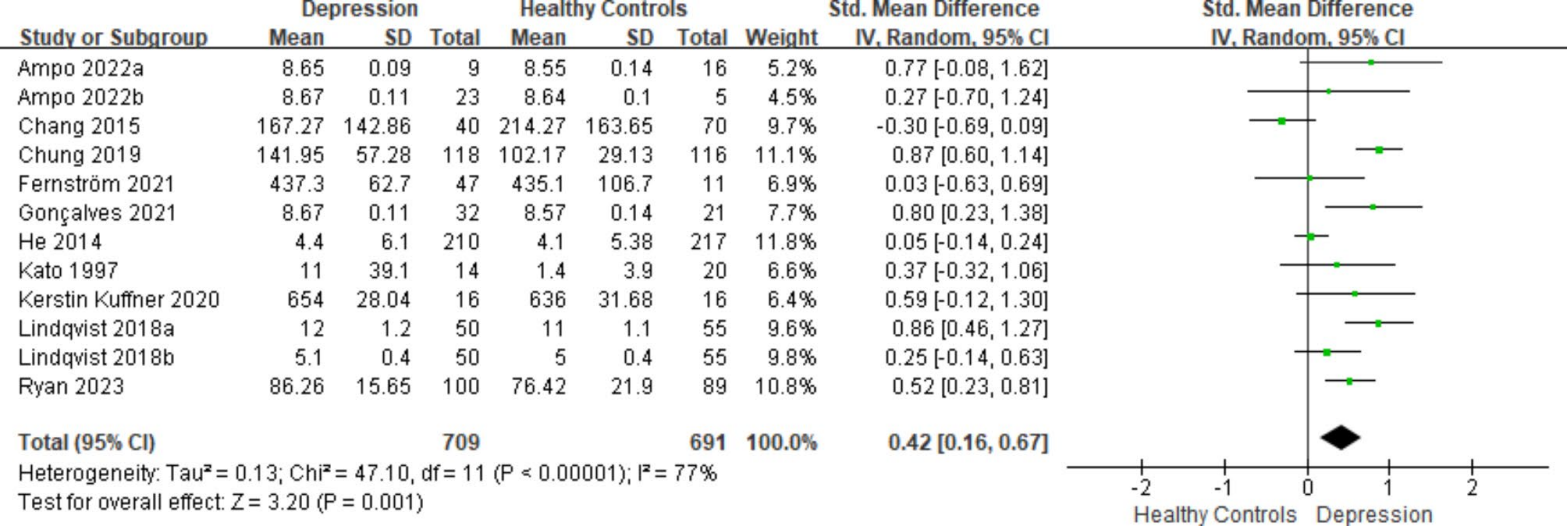
Forest plot displaying the results of the meta-analysis, including all ten articles regarding mtDNA in depression, which comprises twelve comparisons

A substantial variation in mtDNA levels was observed, favoring higher values in individuals with depression when compared to healthy controls. This difference was quantified by a SMD of 0.42 (95% CI: 0.16, 0.67) and an overall effect statistic of Z = 3.20 (*P*-value < 0.05). It is noteworthy that the heterogeneity was substantial (I^2^ = 77%). Please consult Fig. 2 for the comprehensive forest plot illustrating the outcomes of the overall meta-analysis.

### Subgroup analysis

We conducted subgroup analyses based on specimen type (plasma or not plasma), detection method (qRT-PCR, qPCR, or rt-PCR), and geographical location (Asia, Europe, or North America). In terms of specimen type, a significant distinction between individuals with depression and healthy controls was evident in plasma specimens (SMD = 0.52, *P*-value < 0.05), whereas no such difference was observed in other sample types (Fig. 3A). When subgroup analysis was performed based on detection methods, a notable difference between patients with depression (SMD = 0.52, *P*-value < 0.05) and healthy individuals was observed when utilizing qRT-PCR (Fig. 3B). Moreover, in Europe (SMD = 0.48, *P*-value < 0.05) and North America (SMD = 0.69, *P*-value < 0.05), mtDNA levels were significantly higher in individuals with depression than in their healthy counterparts, with no statistically significant variation in Asia (Fig. 3C).

**Fig. 3(A):**
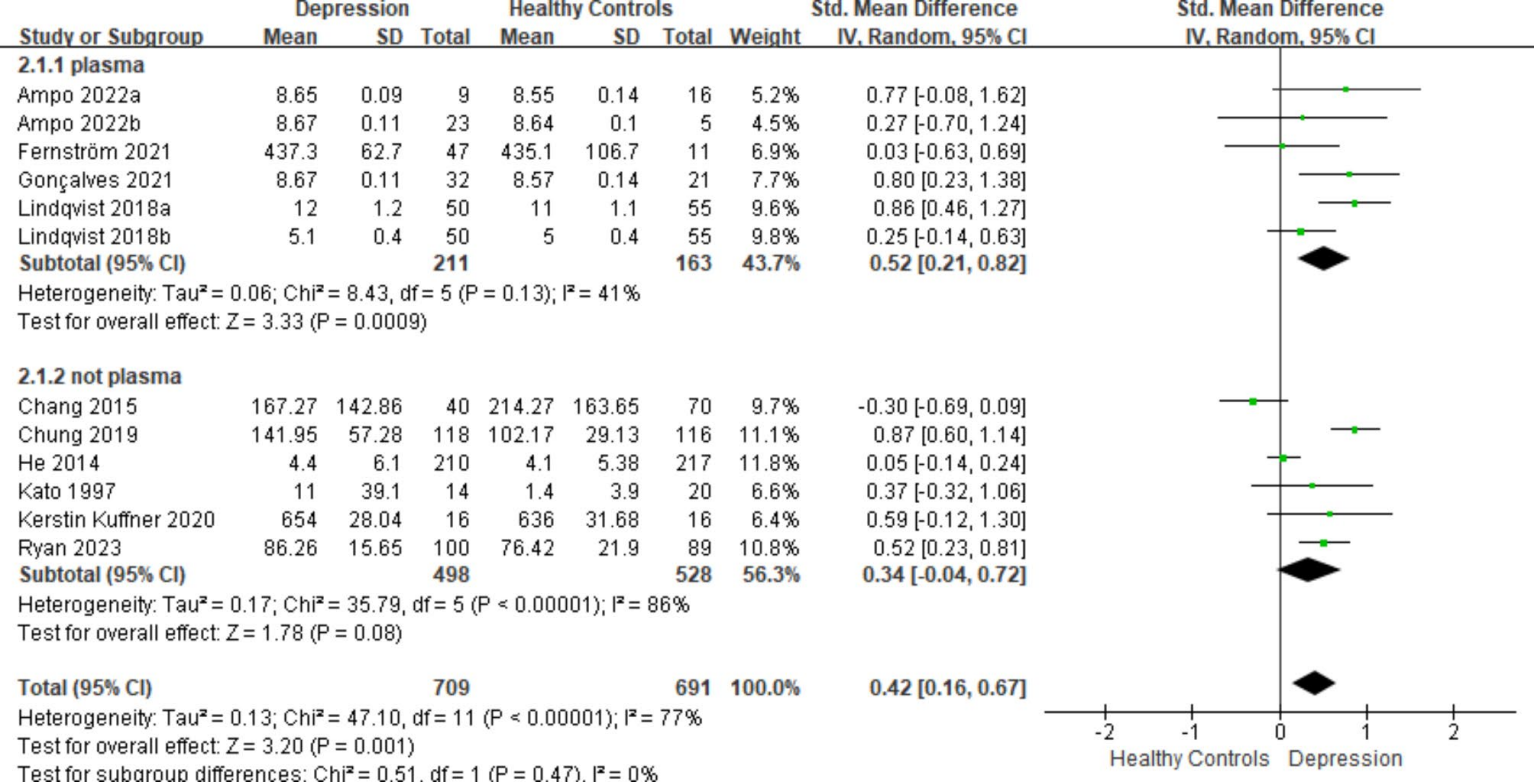
Subgroup analysis categorized according to the specimen type

**Fig. 3(B):**
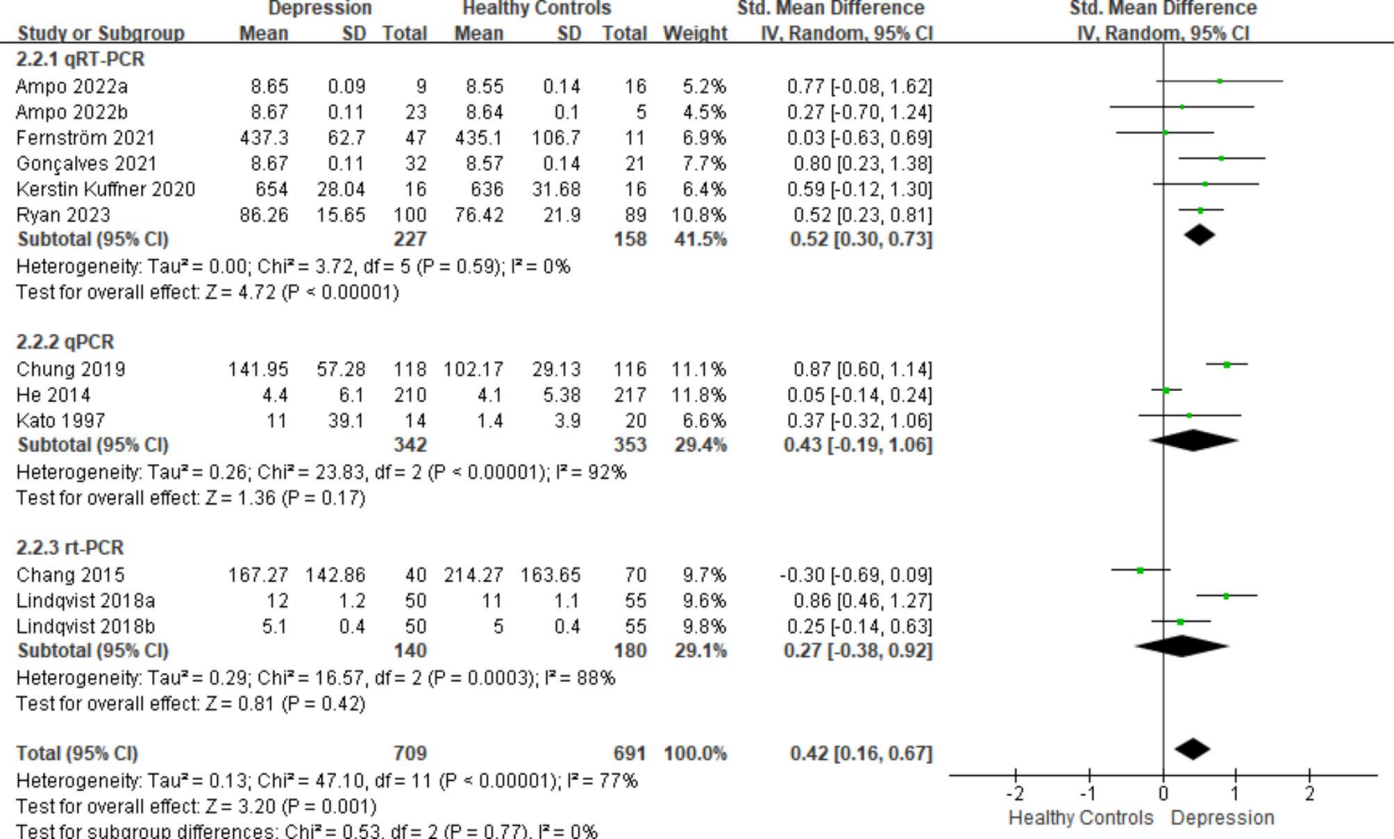
Subgroup analysis categorized according to the detection method

**Fig. 3(C):**
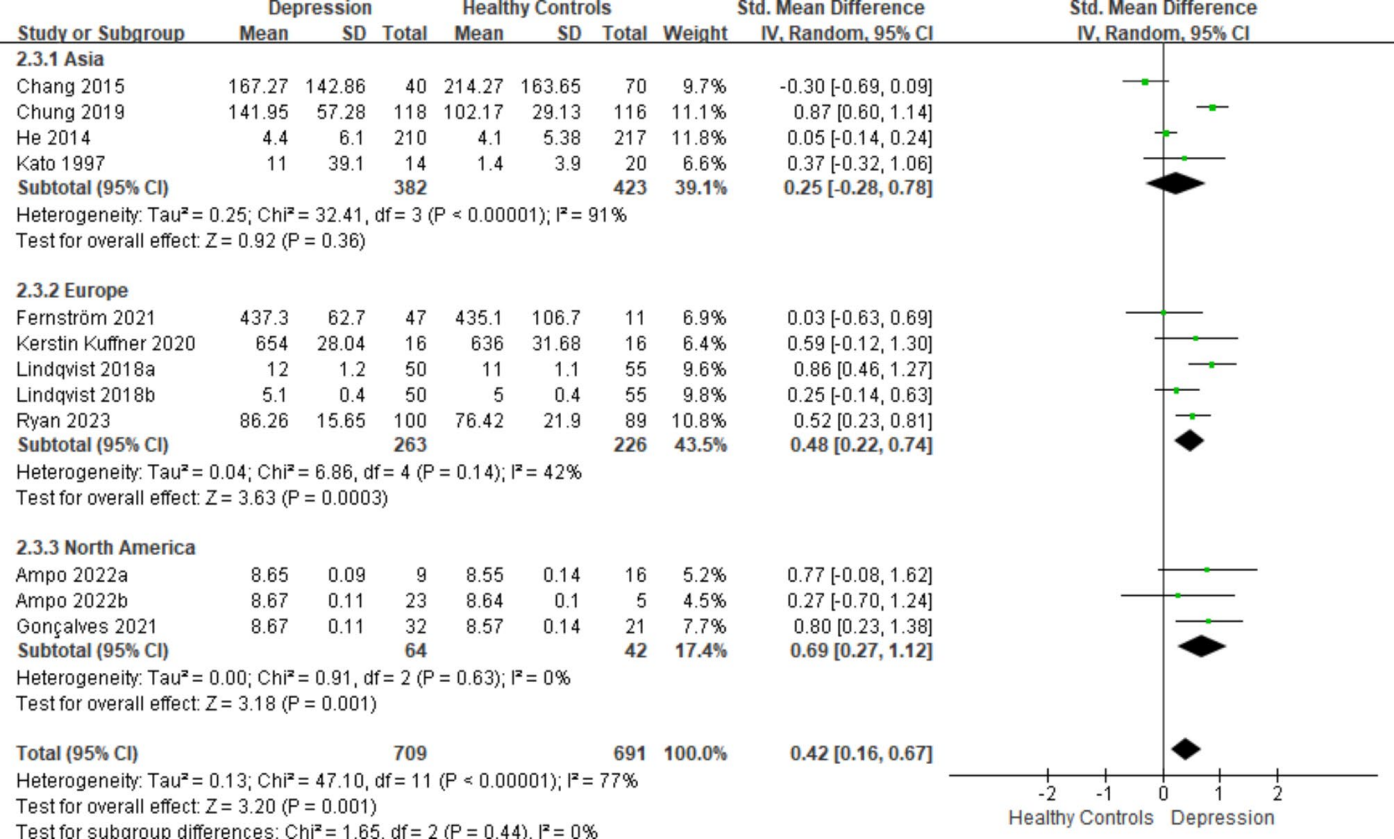
Subgroup analysis categorized according to the geographical location

### Publication bias analysis and sensitivity analysis

**Fig. 4:**
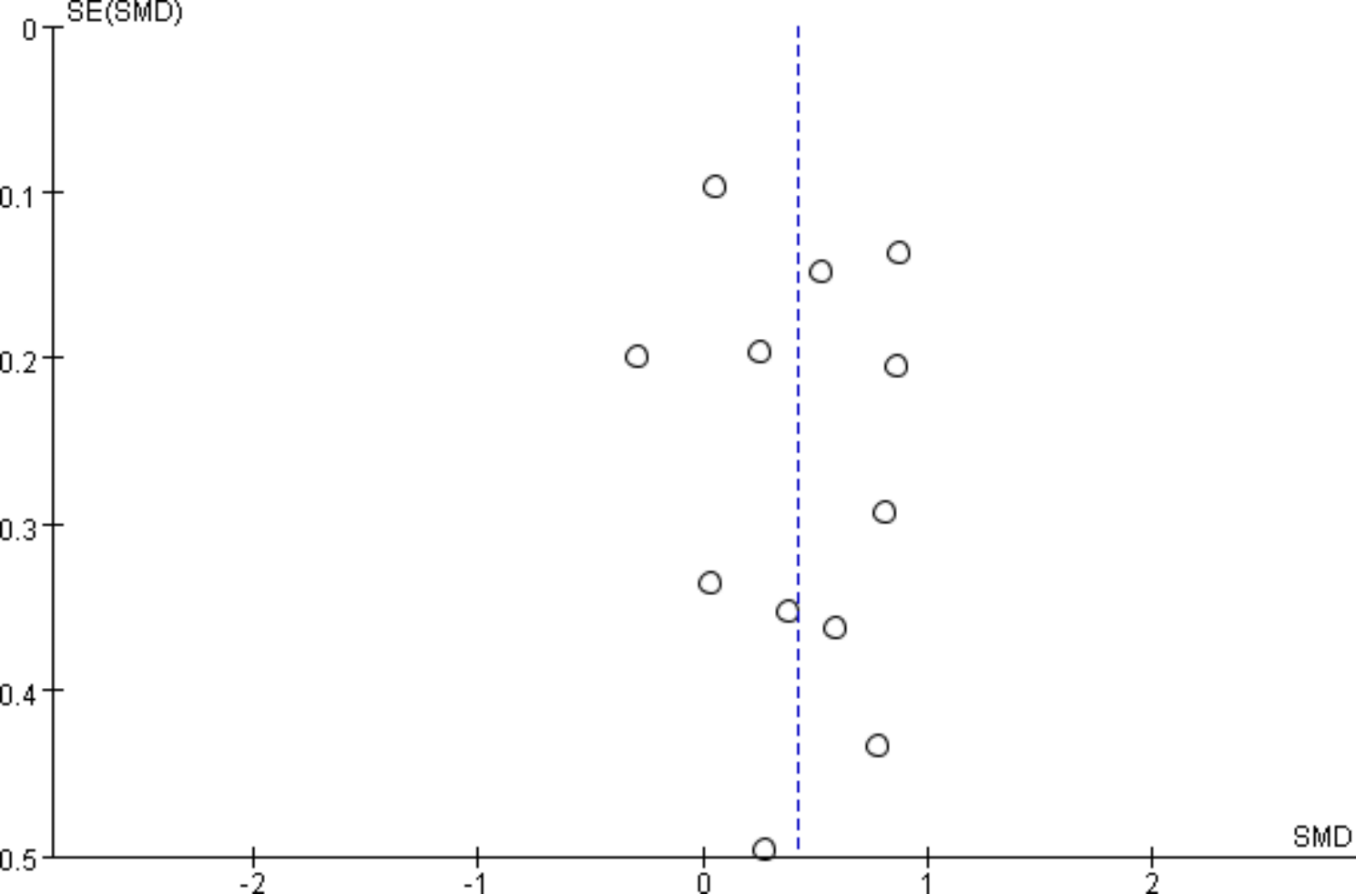
Funnel plot illustrating the studies included in the meta-analysis pertaining to depression

In our study, no evidence of publication bias was found based on the results of both Egger’s regression test (*P*-value = 0.518) and Begg’s continuity corrected test (*P*-value = 1.000). Additionally, there was no apparent asymmetry observed in the funnel plot (Fig. 4). To evaluate the stability of our results, we conducted a sensitivity analysis by systematically removing each study one by one. The results showed no significant impact on the overall findings, reinforcing the stability and reliability of our study outcomes.

## Discussion

### Mitochondrial energy metabolism disorders are involved in depression

Depression poses a significant threat to the mental and physical well-being of individuals, making it one of the most pressing public health challenges worldwide. Alongside the typical clinical signs of depression, such as loss of pleasure and cognitive impairment, there are prominent somatic hypokinetic symptoms like fatigue, insomnia, and appetite loss [27]. Hypodynamic symptoms in depressed patients are closely linked to inadequate mitochondrial ATP production, leading some researchers to suggest that mitochondrial energy metabolism disorders are implicated in depression’s pathogenesis [28]. Mitochondria serve as the central hub for cellular energy metabolism and ROS generation, ensuring their normal function through continuous processes like biosynthesis, division, fusion, and autophagy. Mitochondrial dysfunction includes reduced ATP synthesis, respiratory chain malfunction, structural anomalies, and an excess of apoptosis [29]. Previous research has demonstrated that mitochondrial energy metabolism disruptions are common in both chronic stress animal models and depressed patients [30, 31]. In a study by Gardner et al. [32], examining muscle tissue from depressed patients, they observed a strong connection between low ATP levels and severe physical symptoms. In the hippocampus and gastrocnemius muscle of rats exposed to chronic unpredictable mild stress (CUMS), ATP content, Na/K-ATPase activity, respiratory chain complex I, III, and IV activities were all reduced, and mitochondrial ultrastructure was compromised [33]. The production of a large number of ROS through mitochondrial oxidative phosphorylation can lead to oxidative stress and subsequent mitochondrial dysfunction [34]. When Chen et al. [35] induced depression-like behavior in mice using lipopolysaccharide, they found that mitochondrial oxidative damage was associated with the development of depressive symptoms. In addition, Yuan et al. [33] found significantly more swollen mitochondria, disrupted cristae and broken mitochondrial membranes in hippocampus and gastrocnemius tissue of CUMS rats. Yuan et al. [33], in their study of CUMS rats, noted significantly more swollen mitochondria, disrupted cristae, and broken mitochondrial membranes in the hippocampus and gastrocnemius tissue. It is well-established that maintaining a normal mitochondrial membrane potential (MMP) is a prerequisite for ATP production through oxidative phosphorylation. MMP stability plays a crucial role in preserving normal cell function. Javani et al. [36] discovered a decrease in MMP in the prefrontal cortex of depressed rats, and this condition improved with mitochondrial transplantation. Li et al. [37] found that ginsenoside Rg1 reduced microglial activation and mitochondrial dysfunction, thereby alleviating depression-like behavior through the GAS5/EZH2/SOCS3/NRF2 axis. In conclusion, mitochondrial dysfunction plays a significant role in the pathogenesis of depression, offering new insights into the diagnosis and treatment of this condition.

### MtDNA, mitochondrial energy metabolism disorder, and depression

In addition to the aforementioned factors, including ATP synthesis, oxygen-free radical generation, and changes in MMP, the examination of mitochondrial energy metabolism at the molecular level also includes alterations in mtDNA levels. Serving as the genetic material of mitochondria, mtDNA stands out as the most frequently measured biomarker for assessing mitochondrial dysfunction [38]. However, there is no univocal explanation for variations in mtDNA levels, as both decreases and increases may indicate mitochondrial dysfunction [39]. The number of copies of mtDNA (referred to as mtDNAcn) serves as an effective indicator of a cell’s capacity to produce ATP through mitochondrial oxidative phosphorylation [40]. Consequently, measuring mtDNA levels enables us to evaluate mitochondrial function indirectly. Research suggests that higher mtDNAcn may serve as a marker of poor mitochondrial health or mitochondrial allostatic load, which could potentially account for the observed relationship between higher mtDNAcn and depression [41]. On one hand, oxidative stress can lead to changes in mitochondrial membrane permeability, resulting in increased release of mtDNA [18]. On the other hand, in cases of mitochondrial stress and defective mitophagy, mtDNAs can be released from mitochondria, potentially causing inflammation [42]. Moreover, mitochondrial dysfunction may contribute to depression by promoting oxidative stress and inflammation [43]. A study focusing on cell-free mtDNA suggests that an elevated mtDNAcn may be associated with depression and suicide attempts [44]. Pioneering research by Cai et al. [45] revealed that major depressive disorder was linked to a higher amount of mtDNA in leukocytes from saliva samples and blood. They also discovered altered mitochondrial function in tissues with increased mtDNA [45]. Hence, mtDNA emerges as a promising biomarker for further exploration of its role in depression.

### Discussion of meta-analysis results

Taking into consideration the information presented above, we aimed to provide a comprehensive evaluation to enhance our understanding of altered mtDNA content in depression. In our study, we concentrated on the association between mtDNA levels and depression by conducting a systematic review and meta-analysis of 12 eligible studies, including 709 depressive patients and 691 healthy controls. Among the depressed patients, there were 409 females and 303 males, with females exhibiting a higher risk of depression than males. Gonçalves [19] et al. did not find a significant correlation between ccf-mtDNA concentration and age (r = 0.081, p = 0.56), smoking (measured by number of pack/year; rho = -0.09, p = 0.49). Chung JK [21]et al. found that gender was not associated with mtDNAcn. He [25] et al. also did not find significant differences between age and leukocyte MtDNAcn. However, more high-quality studies are needed to explore whether tobacco, age, and sex have an impact on MtDNA levels in people with depression.

Our study indicated that depressed patients had higher levels of mtDNA compared to their healthy counterparts (SMD = 0.42, 95% CI: 0.16, 0.67). Nonetheless, it is worth noting that published literature has reported conflicting findings. Four studies [17, 19, 21, 23] observed higher mtDNA levels in individuals with depression compared to healthy controls, while eight [18, 20, 22, 23, 25, 26] studies found no significant difference between depressed patients and healthy controls. However, the overall results suggested that depressed patients had higher mtDNA levels than healthy individuals. We attribute these discrepancies to various factors such as differences in age range, detection methods, tissue types, and ethnicity, among others. Heterogeneity was evident in this meta-analysis. Therefore, we conducted subgroup analyses based on specimen type, detection method, and location, as the range of mtDNAcn can be influenced by different DNA isolation and extraction techniques [46]. Moreover, mtDNA is inherently heterogeneous impacted by various environmental and genetic contexts [47]. Furthermore, different tissues or organs within individuals may require varying amounts of mtDNAcn to maintain normal function, resulting in differences in mtDNAcn levels among tissues or organst [38]. Thus, further research is needed to determine the most appropriate sample type (plasma or other samples) and the best detection method for mtDNA assessment.

To assess potential obvious publication bias, we examined funnel plots, Begg’s test, and Egger’s test, all of which did not suggest any noticeable bias. Additionally, our sensitivity analysis reinforced the stability and reliability of our results. It is important to note that the majority of the studies included in our analysis were cross-sectional in design. Therefore, future research should consider initiating more longitudinal studies to evaluate mtDNA levels as a predictive biomarker for depression.

### Strengths and limitations

To the best of our knowledge, this is the first systematic review and meta-analysis of mtDNA levels with a specific focus on depression. Our findings indicate a significant elevation in mtDNA levels in patients with depression when compared to healthy controls, shedding light on the link between mitochondrial dysfunction and depression. Nevertheless, there are certain limitations to consider. Firstly, our results may be affected by the presence of heterogeneity, a common occurrence in many meta-analyses. Secondly, variations in biomarker units and diverse methods of analyzing the same biomarkers could potentially influence the statistical conclusions drawn from this meta-analysis. Thirdly, our study did not explore all the variables affecting mtDNA levels, such as environmental factors and concurrent illnesses. Lastly, due to insufficient numerical information in some studies, we were unable to include them, potentially impacting the results.

## Conclusion

In summary, our systematic review and meta-analysis reveal that mtDNA concentrations in circulating blood samples and skin fibroblasts are elevated in depressed patients compared to healthy controls. This suggests a potential association between higher mtDNA levels and depression. The use of mtDNA as a biomarker for early diagnosis and prognosis of depression and its treatment is a promising avenue. Nonetheless, further research involving high-quality, large-scale studies is necessary for a more in-depth analysis.

### Electronic Supplementary Material

Below is the link to the electronic supplementary material.

Supplementary Material 1: Image corrections

## Data Availability

The original contributions presented in the study are included in the article, further inquiries can be directed to the corresponding authors.
