# Supplementary material for: Association between mitochondrial DNA levels and depression: a systematic review and meta-analysis
Source: BMC Psychiatry. 2023 Nov 22;23:866. doi: 10.1186/s12888-023-05358-8 (PMC10664364; doi:10.1186/s12888-023-05358-8)
Supplement: Supplementary file 1 — Supplementary Material 1: Image corrections [file 12888_2023_5358_MOESM1_ESM.docx]

Dear Editor and Reviewer:

Thanks very much for your kind work and consideration on publication of our paper. We are grateful to you for agreeing to publish our research in your journal. We have changed “Chung 2021” to “Chung 2019” in the figures. Therefore, we need to replace four images（**Figure 2，Figure 3(A)，Figure 3(B)，and Figure 3(C)**）, and the modified images are as follows.

**Figure 2.** **Forest plot displaying the results of the meta-analysis, including all ten articles regarding mtDNA in depression, which comprises twelve comparisons.**

**Figure 3(A). Subgroup analysis categorized according to the** **specimen type.**

**Figure 3(B). Subgroup analysis categorized according to the detection method.**

**Figure 3(C). Subgroup analysis categorized according to the geographical location.**
